# Reevaluation of the Value of Autoparasitoids in Biological Control

**DOI:** 10.1371/journal.pone.0020324

**Published:** 2011-05-25

**Authors:** Lian-Sheng Zang, Tong-Xian Liu, Fang-Hao Wan

**Affiliations:** 1 Institute of Biological Control, Jilin Agricultural University, Changchun, China; 2 Key Laboratory of Applied Entomology, Northwest A&F University, Yangling, Shaanxi, China; 3 Key Laboratory for Biology of Plant Disease and Insect Pests, Institute of Plant Protection, Chinese Academy of Agricultural Sciences, Beijing, China; University of California Merced, United States of America

## Abstract

Autoparasitoids with the capacity of consuming primary parasitoids that share the same hosts to produce males are analogous to intraguild predators. The use of autoparasitoids in biological control programs is a controversial matter because there is little evidence to support the view that autoparasitoids do not disrupt and at times may promote suppression of insect pests in combination with primary parasitoids. We found that *Encarsia sophia*, a facultative autoparasitoid, preferred to use heterospecific hosts as secondary hosts for producing males. The autoparasitoids mated with males originated from heterospecifics may parasitize more hosts than those mated with males from conspecifics. Provided with an adequate number of males, the autoparasitoids killed more hosts than *En. formosa*, a commonly used parasitoid for biological control of whiteflies. This study supports the view that autoparasitoids in combination with primary parasitoids do not disrupt pest management and may enhance such programs. The demonstrated preference of an autoparasitoid for heterospecifics and improved performance of males from heterospecifics observed in this study suggests these criteria should be considered in strategies that endeavor to mass-produce and utilize autoparasitoids in the future.

## Introduction

Aphelinid parasitoids have an outstanding record of success in programs of classical biological control against whiteflies and scale insects [1]. Some exhibit unusual host relationships with males developing on or in different hosts than do the females. Walter called them heteronomous parasitoids [2], which has been universally accepted [3]. Autoparasitoids, the main kind of heteronomous aphelinids, differ from the typical pattern of parasitoid development in which females always develop as primary parasitoids on homopteran or hemipteran hosts (primary hosts), but males develop as hyperparasitoids on their own species or on other primary parasitoids (secondary hosts) [1], [2], [4].

Autoparasitoids occur primarily in the genera *Coccophagus*, *Coccobius*, *Coccophagoides* and *Encarsia* [3]. Some autoparasitoids have been successfully used in biological control in several projects [4], [5]. Their use of immatures of heterospecific parasitoids to produce males results in effects analogous to those observed with intraguild predators [6], [7]. Like intraguild predators, autoparasitoids can consume and kill other primary parasitoids that share the same hosts with them. Because autoparasitoids interact with other conventional parasitoid species with a potential to disrupt pest suppression and eliminate conventional parasitoids, they have at times been considered to be questionable choices in introduction programs [8]–[10]. Several studies on secondary host selection indicate that autoparasitoids prefer heterospecific hosts to conspecific hosts most of the time [11]–[14], which could contraindicate their utility in biological control programs. However, the effects of the autoparasitoid in the context of the population dynamics of the target pest must also be considered.

A series of studies have been conducted worldwide that examine whether autoparasitoids disrupt pest suppression provided by primary parasitoids,. Mills & Gutierrez [8] and Briggs & Collier [15] predicted that autoparasitoids could disrupt pest suppression based on the models they designed. However, Ehler [5] and Heinz & Nelson [16] found that an autoparasitoid and a primary parasitoid together could suppress more pests than could either species used alone. These results appear to be in conflict. Additional studies have also demonstrated that pests can be suppressed with a complex of parasitoids that include at least one species of autoparasitoid and a primary parasitoid under field or greenhouse conditions [5], [7], [16]–[18]. The evidence from the model does not appear to be supported by empirical evidence from the field.

*Encarsia sophia* (Girault & Dodd) ( = *En. transvena*), an autoparasitoid, oviposits female eggs in whitefly nymphs and male eggs externally on female immatures of their own or of other *Encarsia* and *Eretmocerus* species [4]. Recent research demonstrates that the efficiency of *En. sophia* in biological control of whiteflies can be readily manipulated by controlling the duration of food deprivation and effects on mating status [19]–[21]. These parasitoids suppress more whiteflies through parasitism and host feeding than do other commonly used species [22].

In this study, we investigated secondary host selection of the autoparasitoid females on conspecific and heterospecific hosts; compared the suitability of different secondary host species for development of male *En. sophia*, and then explored the performance of males originated from different secondary host species on parasitism of *En. sophia* females. Lastly, we determined the whitefly suppression by *En. sophia* paired with different numbers of males under greenhouse conditions. The goal was to explore the mechanisms affecting heteronomous hyperparasitism in autoparasitoids and the implications this has for development of biological control strategies.

## Materials and Methods

### Parasitoids, whitefly hosts, and host plants

Three species of parasitoids were used in this study: *Encarsia formosa* Gahan, *Eretmocerus melanoscutus* Zolnerowich & Rose and *Encarsia sophia* (Girault & Dodd). *En. formosa* is one of the most commonly used parasitoids for biological control of greenhouse whiteflies. It is a thelytokous endoparasitoid, with females producing no male offspring and laying all its eggs in the whitefly hosts. *Er. melanoscutus* oviposits externally under the nymphal host. This species is a bi-parental primary parasitoid, with both males and females developing in whitefly nymphs. *En. sophia* is an autoparasitoid, and its females are primary parasitoids of whiteflies and males are hyperparasitic, developing on conspecific or heterospecific immatures which are parasitoids of whiteflies [23]. In our previous experiments, we found that the immatures of both *En. formosa* and *Er. melanoscutus* were secondary hosts parasitized by *En. sophia* females. Laboratory colonies of these parasitoids were established using *Bemisia tabaci* biotype B, one of the most serious of global pests [24], on cabbage plants in the Vegetable IPM Laboratory, Texas AgriLife Research and Extension Center in Weslaco, Texas, USA.

Cabbage (*Brassica oleracea* L. var. *capitata*, ‘Golden Acre’) was used as the host plant for *B. tabaci*. Plants were grown in 15-cm plastic pots filled with Metro-Mix 360 growing medium (Sun Gro, Horticulture Distribution Inc., Bellevue, WA, USA) and enclosed in whitefly-proof screen cages (110×80×80 cm). Plants with 3 fully extended true leaves were used in the experiments.

### Secondary host selection

The preference for oviposition of male eggs by *En. sophia* on each stage of their own species and another species, *Er. eremicus*, has been determined by Hunter & Kelly [4]. Their results indicated that *En. sophia* prefers to lay male eggs on late larvae-prepupae of the parasitoids. In this experiment, larvae (3^rd^ instar) of the conspecific *En. sophia* and the heterospecific *En. formosa* and *Er. melanoscutus* were used for examining secondary host selection by *En. sophia* females. In order to obtain the desired stage of secondary hosts, the following procedures were conducted. Thirty female and male adults of *B. tabaci* were introduced onto the lower leaf surface of a cabbage leaf on a potted plant with a leaf clip-on cage (4.0 cm in diameter) for oviposition for 12 h. The development of the eggs was monitored daily, and the nymphs were then monitored daily until they developed to early fourth instars. Then, six mated female parasitoids of *En. sophia*, *Er. melanoscutus*, or six *En. formosa* females were introduced into each clip-cage for oviposition for 12 h, respectively. After parasitoid removal, 20 whitefly adults were introduced into each leaf clip-on cage for oviposition for 6 h (these were the source of healthy whitefly nymphs to be used for host feeding by *En. sophia* females tested in the secondary host selection experiment). The development of parasitoid larvae was monitored daily until they developed to third instar larvae. Cabbage leaf discs prepared with the desired parasitoid stage and healthy whitefly hosts were then used for the following no-choice and paired-choice tests as the secondary hosts for *En. sophia*. All experiments were conducted in an air-conditioned insectary (28±2°C, 70±5% r.h., and a photoperiod of L14∶D10 h).

### No-choice test

Only one species of late larval parasitoid (*En. sophia*, *En. formosa* or *Er. melanoscutus*) was exposed to an *En. sophia* female at a time. The leaf discs with late larval parasitoids and healthy whitefly nymphs as described above were cut out around the leaf clip-on cage's bottom rim. Twenty whitefly nymphs containing the desired stage of larval parasitoids and 20 healthy whitefly nymphs were used on each disk, and all other whitefly nymphs were carefully removed under a stereoscopic microscope using an insect pin. Then, the leaf discs were individually placed on the bottom of a Petri dish (5 cm in diameter and 2.0 cm in depth) covered with a thin layer (0.3–0.5 cm in thickness) of 1.5% agar gel. Two newly-emerged *En. sophia* virgin females (<3 h old) were introduced into each Petri dish. The dishes were inverted to simulate the upside-down natural conditions. After a 48-h exposure time, the survival of parasitoids in each treatment was recorded, and then they were removed. The number of secondary hosts that was parasitized, or fed on by *En. sophia* females were separately counted under a stereoscopic microscope 6 days after parasitoid removal. Parasitoid larvae parasitized by a male larva (“C”-shaped) were easily identified by inspecting the consumed remains of the secondary host [25]. If the secondary hosts were fed on by *En. sophia*, the whitefly nymph's body became flat, and the primary parasitoid larva was visible through the clear cuticle of the host. Each treatment was replicated 25 times.

### Paired-choice test

The late larval instar of two parasitoid species (*En. sophia* vs *En. formosa*, *En. sophia* vs *Er. melanoscutus* or *En. formosa* vs *Er. melanoscutus*) were exposed to *En. sophia* female simultaneously. Half leaf discs (2.5 cm in diameter) had 20 late larval parasitoids of one parasitoid species and 10 healthy whitefly nymphs, and another half leaf disc had same numbers of larvae of another parasitoid species and healthy hosts. Then, two half leaf discs with different secondary parasitoid hosts were placed on the bottom of a Petri dish (5 cm in diameter and 2.0 cm in depth) covered with a thin layer (0.3–0.5 cm in thickness) of 1.5% agar gel. Two newly-emerged *En. sophia* virgin females (<3 h old) were introduced into each Petri dish for 48 h, and numbers of secondary hosts killed by parasitism or host feeding on each half leaf disc in each of the arenas were assessed as described in the no-choice test above. Each treatment was replicated 25 times.

### Development and size of male *En. sophia* originated from different secondary hosts

The leaf discs with 20 late larval parasitoids and 20 healthy whitefly nymphs as described above were individually placed on the bottom of a Petri dish (5 cm in diameter and 2.0 cm in depth) covered with a thin layer of 1.5% agar gel. Five unmated *En. sophia* females (24 h old) were introduced into each Petri dish for hyperparasitism. The dishes were inverted to simulate the upside-down natural conditions. After a 6-h exposure time, the parasitoids in each arena were removed. Each treatment was replicated 20 times. Total numbers of secondary hosts by parasitism or by host-feeding were separately counted under a stereoscopic microscope 5 days after parasitoid removal. Unparasitized parasitoids and healthy whiteflies were removed using an insect pin. The development of male *En. sophia* on different secondary hosts was monitored daily until no parasitoids emerged, and the head width and body length was measured for 30–40 newly-emerged males originated from different secondary hosts.

### Parasitism of *En. sophia* females mated with the males originated from different secondary hosts

One pair of newly-emerged *En. sophia* female and male originated from *En. sophia*, *En. formosa* or *Er. melanoscutus* was introduced onto a cabbage leaf (on a potted plant) with approximately 60 third-instar whitefly nymphs covered by a leaf clip-on cage (4.0 cm in diameter). Every 24 h, the live female and male parasitoids were transferred to a new cabbage leaf bearing a similar number of third-instar whitefly nymphs. During the experiment, only a single male was used to mate with each female. This process lasted for 12 days (preliminary experiments showed parasitism completion for *En. sophia* females in this duration). Host mortality by parasitism or by host feeding of the parasitoid adults on each cabbage leaf was examined under a stereoscopic microscope 6 days after parasitoids were transferred. Twenty pair of *En. sophia* for each treatment was initially used, and the data from 17 to 19 replicates were analyzed because a few females were lost during the experiment.

### Whitefly suppression by *En. sophia* and *En. formosa* under greenhouse conditions

This experiment was conducted in an air-conditioned greenhouse (25–35°C, and 60–90% r.h.). Four potted cabbage plants having 3 fully expanded leaves were placed in each cage (80×45×55 cm) with a glass top and four screened sides. Thirty pairs of newly-emerged *B. tabaci* adults (1∶1 sex ratio) were released into each cage. The development of whitefly immatures was monitored daily. When fourth instar nymphs were first found on the leaves, all introduced whitefly adults were removed using an aspirator, and the plants were re-caged. On the same day, six treatments with different ratios of male to female for *En. sophia* released were conducted in this experiment: (1) 30 females +5 males (females∶males = 6∶1); (2) 30 females +10 males (females∶males = 3∶1); (3) 30 females +15 males (females∶males = 2∶1); (4) 30 females +20 males (females∶males = 3∶2); (5) 30 females +30 males (females∶males = 1∶1); and (6) 30 females of *En. formosa* without *En. sophia*. In this experiment, newly-emerged parasitoids (<6 h old) were used, and all males of *En. sophia* were originated from secondary host *En. formosa*. Each treatment had five replicates. Ten days after parasitoid releases, all leaves with whitefly nymphs from each cage were detached. Total numbers of whitefly nymphs killed by parasitism and host feeding and healthy whitefly nymphs were counted, respectively, as described by Zang & Liu [22].

### Statistical analysis

Parasitism or host feeding by the autoparasitoid *En. sophia* on conspecific and heterospecific hosts under no-choice conditions was analyzed using one-way analysis of variance (ANOVA), and means were separated using Tukey's honestly significantly difference (HSD) test at *P*<0.05 [26]. Numbers of secondary hosts parasitized or fed on by *En. sophia* were transformed to square root to stabilize variance before being subjected to ANOVA. Similarly, one-way ANOVA was used in analyzing the development and size of male *En. sophia* originated from different secondary hosts, parasitism of *En. sophia* females mated with different males originated and whitefly suppression by *En. sophia* at various release ratios of male to female, and means were separated using Tukey's HSD test at *P*<0.05, respectively. Paired *t*-test was used in the analyses of parasitism or host feeding on late larvae of two parasitoid species by *En. sophia* females.

## Results

### Secondary host selection by *En. sophia*

#### No-choice test

**Figure 1:**
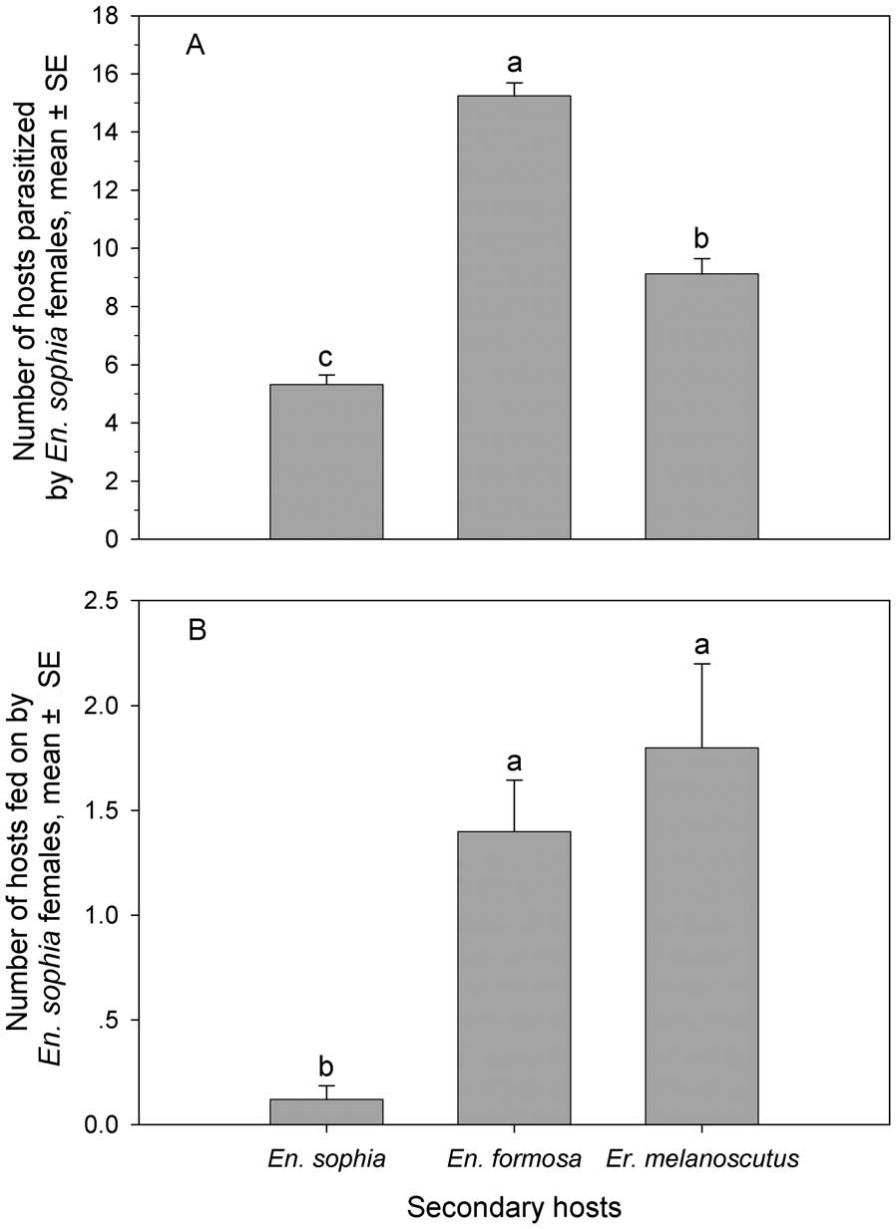
Number of secondary hosts (*En. sophia*, *En. formosa*, *Er. melanoscutus*) parasitized (A) and fed on (B) by two *En. sophia* female adults during 48-h exposure under no-choice conditions. The same letters above bars in each figure indicate that means do not differ significantly (*P*>0.05, Tukey's HSD test).

Parasitism or host feeding by *En. sophia* females on conspecific and heterospecific hosts varied (Fig. 1). *Encarsia sophia* females most preferred to use *En. formosa* as its secondary host, followed by *Er. melanoscutus* and *En. sophia* under no-choice conditions (*F*_2, 72_ = 127.87; *P*<0.0001) (Fig. 1A). The number of *En. formosa* immatures parasitized by one *En. sophia* female in 48 h averaged 7.1, 2.9 and 1.6 fold more than that of *En. sophia* (2.7) and *Er. melanoscutus* (4.6), respectively. *Encarsia sophia* females rarely fed on their own offspring (Fig. 1B). The number of conspecific hosts fed on by *En. sophia* females was significantly fewer than those of heterospecific hosts (*F*_2, 72_ = 10.30; *P* = 0.0001), *Er. melanoscutus* and *En. formosa* with no difference between the latter.

#### Paired choice test

**Figure 2:**
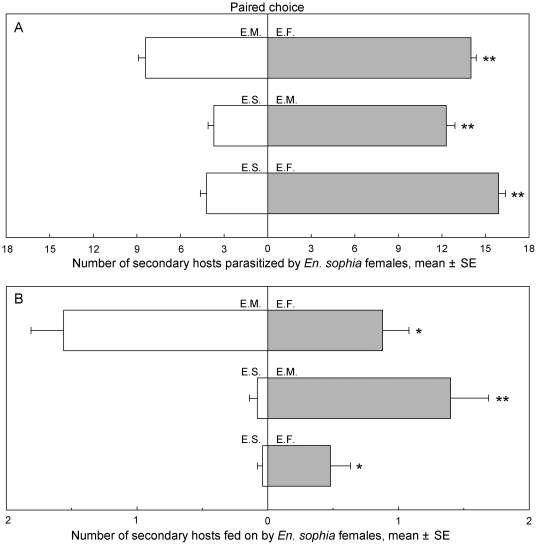
Number of secondary hosts parasitized (A) and fed on (B) by two *En. sophia* female adults during 48-h exposure under paired choice conditions. The paired bars with an ‘*’ or ‘**’ indicate that the means differ significantly at *P*<0.05 or *P*<0.01 (paired *t*-test), respectively. Secondary hosts∶ E.S. = *En. sophia*, E.F. = *En. formosa*, E.M. = *Er. melanoscutus*.

*Encarsia sophia* exhibited different host selection on different secondary host species under paired choice conditions (Fig. 2). When *En. sophia* was offered in combination with heterospecific species, *En. sophia* females significantly preferred parasitizing *En. formosa* or *Er. melanoscutus* compared to its own species (15.9 *En. formosa* vs 4.2 *En. sophia*; 12.3 *Er. melanoscutus* vs 3.7 *En. sophia*. *t* = 11.89−18.07, *P*<0.0001). When *En. formosa* and *Er. melanoscutus* were offered simultaneously, *En. sophia* females significantly preferred to parasitize *En. formosa* (14.0 vs 8.4, *t* = 9.10, *P*<0.0001) (Fig. 2A). When *En. sophia* was presented in combination with heterospecific species, *En. sophia* females significantly fed on fewer conspecific hosts than heterospecific hosts, *En. formosa* and *Er. melanoscutus* (*t* = 2.76−4.41, *P* = 0.0101−0.0002). However, when two heterspecific hosts were presented simultaneously, *En. sophia* females significantly fed on more *Er. melanoscutus* than *En. formosa* (*t* = 2.14, *P* = 0.0378) (Fig. 2B).

### Development and size of male *En. sophia* originated from different secondary hosts

Male development time was significantly different in different secondary host species (Table 1). *En. sophia* males developed fastest on *En. formosa*, followed by *En. sophia* and *Er. melanoscutus*. *En. sophia* males had greatest proportion of emergence on their own species. There was no difference with *En. formosa*, but significantly higher than on *Er. melanoscutus*. Males originated from different secondary host species differed significantly in size. The largest males were obtained from *En. formosa* (Table 1).

**Table 1:** Comparisons of development time, proportion of emergence and size of males originated from different secondary hosts.

Secondary host species	Male development time (days) ± SE	Proportion of male emergence ± SE	Size of male	
			Head width (mm) ± SE	Body length (mm) ± SE
*En. sophia*	12.1±0.1 b	0.92±0.03 a	0.225±0.038 ab	0.537±0.091 b
*En. formosa*	11.5±0.1 c	0.84±0.03 ab	0.231±0.039 a	0.565±0.096 a
*Er. melanoscutus*	12.8±0.1 a	0.75±0.03 b	0.221±0.037 b	0.517±0.087 b
	*F*_2, 55_ = 28.07*P*<0.0001	*F*_2, 55_ = 7.79*P* = 0.0011	*F*_2, 102_ = 6.55*P* = 0.0021	*F*_2, 102_ = 14.35*P*<0.0001

### Parasitism of *En. sophia* females mated with males originated from different secondary host species

**Figure 3:**
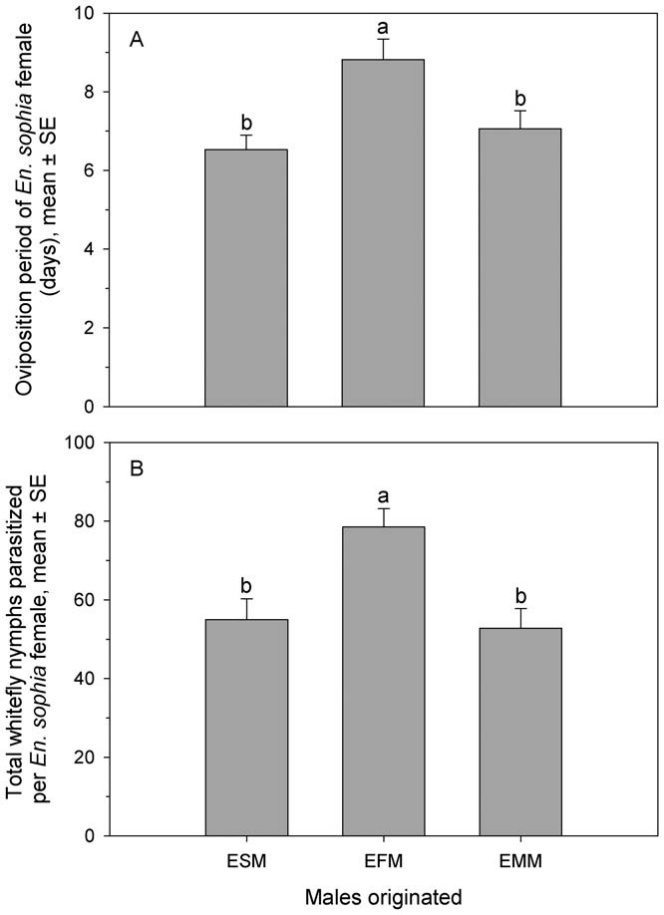
Oviposition period (A) and total whitefly nymphs parasitized (B) by *En. sophia* female mated with male originated from different secondary hosts. The same letters above the bars in each figure indicate that means do not differ significantly (*P*>0.05, Tukey's HSD test). ESM, EFM and EMM indicated that males from *En. sophia*, *En. formosa* and *Er. melanoscutus*, respectively.

*Encarsia sophia* females had different oviposition periods and parasitized different numbers of whitefly nymphs throughout their lifespan when they were mated with males originated from different secondary host species (Fig. 3). Females mated with males originated from *En. formosa* had longer oviposition periods (*F*_2, 50_ = 7.22; *P* = 0.0018) and parasitized more whitefly nymphs (*F*_2, 50_ = 7.87; *P* = 0.0011) than those mated with males from *En. sophia* and *Er. melanoscutus* with no difference between them.

### Whitefly suppression by *En. sophia* and *En. formosa* under greenhouse conditions

**Figure 4:**
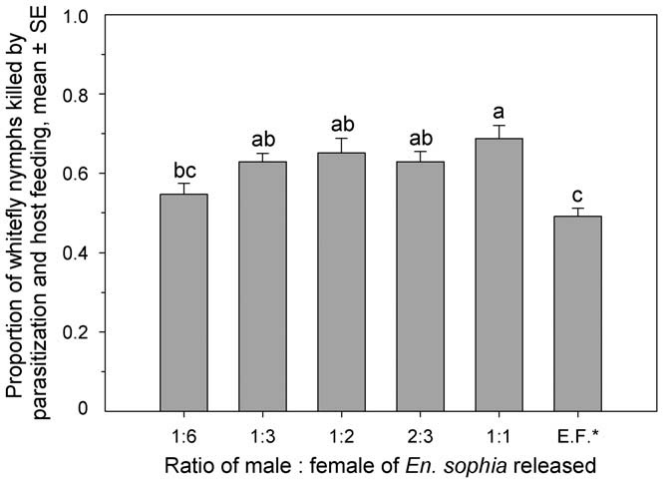
Proportion of total whitefly nymphs killed due to parasitism and host feeding by *En. sophia* with different released ratio of male: female and *En. formosa*. The same letters above bars in each figure indicate that means do not differ significantly (*P*>0.05, Tukey's HSD test). E.F. - *En. formosa*.

The availability of males significantly affected the efficiency of whitefly suppression by *En. sophia* (*F*_4, 20_ = 3.07; *P* = 0.0402) (Fig. 4). *Encarsia sophia* released at a male to female ratio of 1∶1 caused the largest proportion of whitefly nymph mortality by parasitism and host feeding among all treatments of parasitoid releases. Generally, *En. formosa* caused significantly lower proportion of whitefly nymph mortality than *En. sophia* released at all male to female ratios except at 1∶6, with no difference between them.

## Discussion

The attack on heterospecific competitors through parasitism and host feeding by autoparasitoids represents a mechanism of interference similar to intraguild predation [9]. Similar to intraguild predators, the autoparasitoid females consume and kill both their competitors and a shared host [3]. As for threatening the establishment and survival of other parasitoid species, the relaxed predation induced by autoparasitoids possibly results in temporal outbreaks of some pests [10], and they are also predicted to have the potential to disrupt pest suppression based on the models established [8], [15]. However, numerous reports show that autoparasitoids have been successfully used for biological control of whiteflies, blackflies, scales and midges [5], [7], [16], [27].

Several studies on hyperparasitism behavior of autoparasitoids on secondary hosts have been conducted. In some *Encarsia* species, such as *En. pergandiella* Howard and *En. transvena* ( = *En. sophia*), no parasitism preference has been found between conspecific and heterospecific secondary hosts [4], [13]. However, *En. tricolor* Forster and *En. smithi* (Silvestri) exhibit parasitism preference for heterospecific hosts more than conspecific hosts [11], [12], [14], [28]. The present study indicates that *En. sophia* females prefer to use heterospecifics as secondary hosts, and particularly prefer to parasitize *En. formosa* under no-choice or choice conditions (Figs. 1, 2). The inconsistent results with Hunter & Kelly [4] possibly result from different host insects (*B. tabaci* vs *Trialeurodes vaporariorum*), host plants (cabbage vs green bean) and heterospecific secondary hosts (*En. formosa*, *Er. melanoscutus* vs *Er. eremicus*) in the two experimental systems. Certainly, another possible reason for differences is connected with virgin *En. sophia* females our study and mated females used in theirs. *Encarsia sophia*, a destructive host feeder, causes significant whitefly mortality by host feeding, equivalent to that caused by parasitism [22]. The results of secondary hosts killed by host feeding indicated an *En. sophia* female could feed on about 1–2 heterospecific hosts in 24 h, and hardly fed at all on conspecific hosts. The conspecific hosts are rarely fed on by *En. sophia* females; this may be related to the narrow window of vulnerability [4] and perhaps self-discriminatory behavior. Besides hyperparasitism, autoparasitoids also can feed on secondary hosts with a significant difference occurring between heterospecifics and conspecifics.

The autoparasitoid females might attack developing conspecific hosts and produce their males in future. Most studies demonstrated that there was host stage suitability for development of autoparasitic males [4], [29]–[31]. Generally, the host stages of late larvae to prepupae were suitable for development. Facultative autoparasitoids hyperparasitize conspecifics and heterospecifics [2], thus their males may have different secondary host origins. Avilla & Copland [30] investigated secondary host suitability of *En. tricolor* and *En. formosa* for development of *En. tricolor* males. Their results showed that *En. tricolor* males developed faster on *En. formosa* than on their own species when the suitable host stages were offered. Similarly, our study indicated that *En. formosa* was the most suitable host for male development of *En. sophia* with the largest individuals and highest proportion of emergence in the shortest period of time (Table 1). The high suitability of heterospecific hosts hints that facultative autoparasitism is not only a trait that self-regulates population densities and confers stability on host and parasitoid population but is a means of enabling coexistence with competitors in natural conditions [30]. Preference for parasitizing heterospecific hosts by an autoparasitoid may allow them to produce more males that contribute to their own population expansion. Further, the possibility for males to develop on females of their own species can be envisaged as a strategy for maintaining population persistence when there are no competing species in the same habitat. These are expected to be factors that affected autoparasitoid evolution.

As for facultative autoparasitoids, males may have different origins, conspecific or heterospecific hosts. In this study we found that the males originated from different host species affected parasitism of autoparasitoid females. Our results demonstrated that there was a significant difference in parasitism of *En. sophia* females on whiteflies mated with males from different secondary hosts (Fig. 3). Generally, *En. sophia* females had a longer oviposition period and parasitized more whiteflies when they were mated with males originated from *En*. *formosa*. In addition, *En. sophia* females mated with males from *Er. melanoscutus* and its own species expressed similar parasitism of whiteflies. The females of some parasitoid species such as *Cotesia glomerata* (L.), mated with haploid males, could produce more daughters than females mated with diploid males [32]. The present study indicated that males originated from different hosts also might influence the reproduction of autoparasitoid females. West & Rivero [33] estimated the factors that limit reproduction in parasitoids from autoparasitoid sex ratio data. Our results suggest that secondary host species may influence their reproduction as well. The special reproductive biology in autoparasitoids should be considered when developing theoretical models that predict the optimal oviposition strategy. Many authors suggest that autoparasitoids may be dominant more often than primary parasitoids in parasitoid communities [1]. The autoparasitoids may produce more males when they coexist with other parasitoid species. Consequently, the mated autoparasitoids using primary hosts may produce more females, which further contributes to the expansion of their own population. Our results present evidence to support the notion that autoparasitoids are the dominant species in the majority of natural parasitoid complexes [1].

The literature addressing the efficiency of most autoparasitoids as biological control agents to suppress primary hosts is documented in Table 2. In most cases, the addition of a facultative autoparasitoid such as *Coccophagus lycimnia* (Walker), *En. smithi* (Silv.) and *En. tricolor*, exhibit the potential to disrupt primary hosts. However, in several cases, primary hosts can be successfully suppressed by an autoparasitoid alone or with a parasitoid complex that includes at least one autoparasitoid and a primary parasitoid that is attacked by it in biological control programs (Table 2). Several autoparasitoids such as *Zatropis capitis* Burks, *En. pergandiella* and *En. sophia*, may at times suppress more pests in combination with primary parasitoids than either species used alone [5], [7], [16]. Our results indicate that males played a principal role in suppressing pests by autoparasitoids. When the ratio of male to female of *En. sophia* was not less than 1∶3, the autoparasitoid exhibited superior bio-control efficacy on whiteflies than did the primary parasitoid, *En. formosa* (Fig. 4). The preference of autoparasitoids to parasitize heterospecific hosts results in producing more males when they coexist with other primary parasitoids. On the other hand, the autoparasitoid females may parasitize more or similar number of pests when they mate with the males from heterospecific hosts. All of these may offset the adverse effects from autoparasitoids that consume primary parasitoids. The present study provides evidence to explain why an autoparasitoid in combination with a primary parasitoid that is attacked by it may suppress more insect pests. Our results could alleviate some fears that releasing autoparasitoids may disrupt parasitoid diversity and pest suppression. Presently many conventional primary parasitoids (e.g. *En. formosa* and *Er. erimicus*) have been commercially produced and widely applied in pest management. However, due to the biological and behavioral features, mass-production of autoparasitoids has been constrained for seasonal inoculative or inundative releases [34]. The apparent preference for heterospecific hosts and satisfactory performance of males from heterospecifics reveals a direction to mass-produce autoparasitoids in future.

**Table 2:** The efficiency on suppression of primary hosts by autoparasitoids alone or in combination with primary parasitoids in biological control programs.

Parasitoid species	Male development^a^	Primary hosts	Evaluation as biological control agent^b^	Reference
*Coccophagoides utilis* Doutt	C	*Parlatoria oleae* (Colvee)	+ +	36
*Coccophagus gurneyi*Compere	H-C	*Pseudococcus calceolariae* (Mask.)		8, 37
*C. lycimnia* (Walker)	H-C	*Coccus pseudomagnoliarum* (Kuwana)	+ −	38
		*Toumeyella pini* (King)	+ +	39
*C.* sp. nr *gurneyi*	H	*Lantana montevidensis* (Spreng)	+	40
*C. cowperi* Girault	H-C	*Pulvinariella mesembryanthemis* (Vallot)	+	29
*Encarsia* sp.	H-C	*B. tabaci* (Gennadius)	+ −	8
*Encarsia* sp.	C	*B. tabaci*	+ +	8
*En. bimaculata*(Heraty & Polaszek)	H-C	*B. tabaci*	+	41
*En. lahorensis* (Howard)	H-C	*Dialeurodes citri* (Ashmead)	+ +	42
*En. opulenta* (Silv.)	C	*Aleurocanthus woglumi* Ashby	+ +	17, 35
*En. pergandiella* Howard	H-C	*B. argentifolii* Bellows&Perring	+ + +	16
*En. perniciosi* (Tower)	H-C	*Quadraspidiotus perniciosus* (Comstock)	+	8
		*Aonidiella aurantii* (Mask.)	+ +	43
*En. smithi* (Silv.)	H-C	*A. spiniferus* (Quaintance)	+	8
		*A. woglumi* Ashby	+ −	35
*En. sophia* (Girault&Dodd)	H-C	*B. argentifolii*	+ + +	7
		*Parabemisia myricae* (Kuwana)	+ +	8
*En. sublutea* Silv.	H-C	*B. tabaci*	+ +	44
*En. tricolor* Forster	H-C	*Aleyrodes proletella* L.	+ −	45
*Physcus seminotus* Silv.	H-C	*Aulacaspis tegalensis* (Zhnt.)	+ +	46
*P. subflavus* Annecke&Insley	H-C	*A. tegalensis* (Zhnt.)	+	47
*Zatropis capitis* Burks	H-C	*Rhopalomyia californica* Felt	+ + +	5

a C: Males develop on conspecifics only; H: Males develop on heterospecifics only; H–C: Males may develop on heterospecifics or on conspecifics.

b +: A potential biological control agent; + +: No disruption on the suppression of primary hosts; + −: Disrupt suppression of primary hosts in combination with primary parasitoids; + + +: Suppress more primary hosts in combination with primary parasitoids.
